# Neighborhood support as a protective factor for cognition: Associations with sleep, depression, and stress

**DOI:** 10.1002/alz.70940

**Published:** 2025-11-25

**Authors:** Ramkrishna K. Singh, Semere Bekena, Yiqi Zhu, Jean‐Francois Trani, Anthony Briggs, Omonigho M. Bubu, Brendan P. Lucey, Beau M. Ances, Ganesh M. Babulal, for the Aging Adult Brain Connectome (AABC) study team

**Affiliations:** ^1^ Department of Neurology Washington University School of Medicine St. Louis Missouri USA; ^2^ Department of Psychology University of Johannesburg Johannesburg South Africa; ^3^ Institute of Public Health Washington University School of Medicine St. Louis Missouri USA; ^4^ School of Public Health Washington University in St. Louis St. Louis Missouri USA; ^5^ Departments of Psychiatry Neurology and Population Health and NYU Neuroscience Institute NYU Grossman School of Medicine New York New York USA; ^6^ NYU Alzheimer's Disease Research Center NYU Grossman School of Medicine New York New York USA

**Keywords:** aging population, cognitive aging, cognitive resilience, dementia risk, depression, neighborhood social cohesion, Preclinical Alzheimer's Cognitive Composite, psychosocial stress, resilience mechanisms, sleep quality

## Abstract

**INTRODUCTION:**

Sleep, depression, stress, and neighborhood support are independently linked to cognition, but how these factors interact when sleep quality is poor remains understudied.

**METHODS:**

We analyzed cross‐sectional baseline data from 233 adults aged ≥ 65 years in the Aging Adult Brain Connectome study. Sleep quality, depressive symptoms, stress, and neighborhood support were assessed with validated scales, and cognition was measured using the Preclinical Alzheimer's Cognitive Composite (PACC). Models tested two‐ and three‐way interactions, adjusting for sociodemographics.

**RESULTS:**

Poor sleep quality was associated with lower PACC scores (β = −0.57, *p* = 0.002). This association was even more pronounced in older adults who also had depressive symptoms (β = −0.09, *p* < 0.001) or increased stress (β = −0.31, *p* < 0.001). This effect was attenuated by greater neighborhood support (interaction estimates 0.007–0.021, all *p* ≤ 0.014).

**DISCUSSION:**

Poor sleep quality was associated with lower cognition, compounded by psychosocial burden and buffered by neighborhood support.

**Highlights:**

- Poor sleep quality worsened late‐life cognitive performance in older adults.
- Depressive symptoms and stress further worsened the effect of poor sleep on cognitive performance.
- Neighborhood support buffered negative sleep–psychosocial impacts on cognitive performance.

## 1 BACKGROUND

As populations age worldwide, promoting cognitive health and reducing dementia risk have emerged as central public health priorities. Identifying modifiable biological and psychosocial risks is critical to extending cognitive health span and delaying the onset of dementia.^1^ Among these risks, sleep disturbance and psychosocial burden, including depression and chronic stress, are especially common in older adults and increasingly recognized in accelerated cognitive decline.^2^, ^3^ This evidence highlights psychosocial burden as a modifiable risk state that interacts with other exposures, such as sleep, to shape late‐life cognitive outcomes.^4^

To capture these complex, interrelated influences, the exposome framework provides a comprehensive lens to understand how cumulative environmental, behavioral, and biological exposures interact across the life course. It has been applied to understand brain health and cognitive functioning.^5^ By situating neighborhood, psychosocial, and biological influences within the exposome, research can more precisely map how structural, social, and individual pathways converge to affect late‐life cognitive performance.^6^ This framework highlights the value of examining interconnected influences rather than isolated risks.

Sleep may represent one such pathway through which psychosocial risks exert their influence on cognitive function. Sleep problems are highly prevalent in late life and consistently linked to cognitive deficits and increased dementia risk.^7^ Large population‐based studies show that insomnia symptoms, abnormal sleep duration, and sleep medication use predict dementia independent of demographic and health covariates.^7^, ^8^ In longitudinal studies, sleep disturbance is also associated with trajectories of cognitive decline and with biomarkers of preclinical Alzheimer's disease (AD).^9^, ^10^ These converging findings highlight sleep as a viable clinical target with potential downstream cognitive benefits. Yet sleep rarely acts in isolation, and its consequences often depend on other co‐occurring psychosocial risks.

**RESEARCH IN CONTEXT**

1. **Systematic review**: Prior studies have linked poor sleep quality, depressive symptoms, stress, and low social support to worse cognitive performance, but most have examined these factors independently. Few studies have investigated how psychosocial burden and neighborhood support interact to influence the impact of sleep quality on cognition.
2. **Interpretation**: In this cross‐sectional study of community‐dwelling older adults, poor sleep quality was associated with worse cognitive performance. These adverse associations were magnified by the presence of depressive symptoms and/or stress but were attenuated by stronger neighborhood support. This pattern suggests that psychosocial burdens can intensify the negative impact of poor sleep quality, while social support can help mitigate it.
3. **Future directions**: Sleep disturbance and mood disorders are modifiable risk factors for cognitive decline. Longitudinal research is needed to clarify causal pathways, and intervention studies should evaluate whether improving these factors, alongside strengthening supportive neighborhood environments, can mitigate cognitive decline. This approach is consistent with the Lancet Commission framework, which emphasizes the importance of addressing modifiable risk factors in dementia prevention.

One such risk is depression, which is common in older adults and independently elevates dementia risk.^11^ Depression more than doubles dementia risk across the lifespan.^11^ Beyond depression, chronic stress and broader psychosocial distress have also been linked to mild cognitive impairment (MCI) and dementia, with additive or synergistic risks when stress and depression co‐occur.^3^, ^12^ Thus, psychosocial burden is not merely a correlate of cognitive decline but a modifiable risk state that may interact with sleep to shape outcomes. To comprehensively understand these dynamics, analyses require the inclusion of social contexts in which adults live.

Neighborhood environments influence exposure to stress, sleep health, and access to informal support.^13^ Broader structural and social determinants, including racialized inequities, significantly contribute to dementia risk and resilience.^14^, ^15^ In the United States, poorer neighborhood quality, including greater disorder and lower cohesion, has been linked to more insomnia symptoms, worse sleep, higher depressive symptoms, and poorer health in adults > 50 years.^16^ In contrast, more cohesive neighborhoods are associated with better sleep and psychosocial well‐being, partially through factors such as depression and perceived control.^17^ Indeed, social support has been shown to confer cognitive benefits, with reviews indicating that social relationships and support promote better global cognition and episodic memory in older adults.^18^ Taken together, these findings underscore that neighborhood context is deeply intertwined with sleep, psychosocial well‐being, and resilience in later life.

Building on the existing literature, this study examined the association between self‐reported sleep disturbance and late‐life cognition and investigated whether these associations were amplified by psychosocial burden (depressive symptoms or stress) and buffered by neighborhood support. We hypothesized that poor sleep quality would be associated with lower cognitive performance, that this adverse effect would be stronger under higher psychosocial burden, and that greater neighborhood support would mitigate these combined negative associations. By modeling two‐ and three‐way interactions among sleep, psychosocial risk, and neighborhood support, and comparing outcomes, this study provides a targeted test of a multilevel resilience framework for cognitive aging.

## 2 METHODS

### 2.1 Study design and participants

This cross‐sectional study used baseline data from the Aging Adult Brain Connectome (AABC) study, a multi‐site longitudinal cohort conducted at Washington University in St. Louis, Harvard/Massachusetts General Hospital in Boston, University of California Los Angeles David Geffen School of Medicine, and the University of Minnesota‐Twin Cities. Participants were recruited through academic health systems and community outreach at the four sites. The cohort reflects a volunteer sample of community‐dwelling adults aged ≥ 36 years but is not population based. As part of the parent study, participants completed clinical, cognitive, and biomarker assessments every 2 years. For the current analysis, data from the baseline time point were used. We focused on participants ≥ 65 years because late life represents the period of highest risk for cognitive decline, when sleep, mood, and psychosocial factors may exert the strongest influence on cognition.

Eligible participants were physically healthy, fluent in English, and able to travel to the study site. Sociodemographic data such as age, sex, and education level were collected. Older adults with any psychiatric (including major depression) or neurological disorders, severe medical illnesses limiting life expectancy (e.g., advanced cancer), recent substance abuse, current use of medications known to affect cognition, or a history of traumatic brain injury involving loss of consciousness were excluded. Cognitive status was assessed cross‐sectionally using standardized test scores (Montreal Cognitive Assessment [MoCA] and Preclinical Alzheimer's Cognitive Composite [PACC]). Lower scores on brief screening tools can occur for various reasons, including age, education, or temporary factors such as fatigue, and should be interpreted with caution.

The study protocol was approved by the institutional review board at Washington University in St. Louis (IRB #: 202201003‐1001) and complied with the Declaration of Helsinki. Written informed consent was obtained from all participants. This study adheres to the Strengthening the Reporting of Observational Studies in Epidemiology (STROBE) guidelines for cross‐sectional studies.

### 2.2 Cognitive assessments

Cognitive performance was assessed using the PACC, comprising tests of episodic memory, processing speed, executive function, and semantic memory.^19^ Episodic memory was assessed with the Rey Auditory Verbal Learning Test (RAVLT), processing speed and executive function with the Trail Making Test Parts A and B (TMT‐A, TMT‐B),^19^ and semantic memory with the National Institutes of Health Toolbox Picture Vocabulary Test.^20^ MoCA was also administered to evaluate global cognitive function.^21^

### 2.3 Sleep assessments

Sleep quality was assessed using the Pittsburgh Sleep Quality Index (PSQI), a validated self‐report questionnaire with scores ranging from 0 to 21, with higher scores indicating poor sleep quality.^22^ For analysis, the PSQI was modeled both as a continuous variable (global score) and as a categorical variable, with scores ≤ 7 classified as indicating good sleep quality and scores > 7 classified as indicating poor sleep quality.^23^, ^24^, ^25^ This dual operationalization allowed us to evaluate both graded and threshold‐based associations among sleep quality, psychosocial factors, and cognitive performance.

### 2.4 Assessment of psychosocial factors

#### 2.4.1 Depressive symptoms assessments

Depressive symptoms were assessed using the 20‐item Center for Epidemiologic Studies Depression Scale (CES‐D). Items were rated on a 4‐point Likert scale (0 = rarely or none of the time to 3 = most or all of the time), with four positively worded items reverse scored. Total scores range from 0 to 60, with higher values indicating greater depressive symptomatology. A cutoff of ≥ 16 is commonly used to identify elevated symptoms, though the scale is also applied as a continuous measure in epidemiologic research to capture subclinical symptoms.^26^

#### 2.4.2 Ongoing chronic stress assessments

Chronic stress was measured using the eight‐item Ongoing Chronic Stressors Scale (OCSS). The scale assessed the presence and intensity of common stressors (e.g., health problems, financial strain, housing difficulties, caregiving demands) experienced over the past year or longer. Each item is rated from “no, didn't happen” to “yes, very upsetting,” with higher total scores reflecting greater levels of ongoing stress.^27^

#### 2.4.3 Neighborhood social cohesion assessments

Neighborhood social cohesion was measured using the social cohesion subscale, which assessed perceptions of trust, mutual support, and connectedness among neighbors. Higher scores indicate stronger perceived cohesion. ^28^, ^29^

#### 2.4.4 Psychometric reliability assessment

Internal consistency was assessed for scales with available item‐level data. Social cohesion showed strong reliability (α = 0.90), while stress (α = 0.65) and sleep (α = 0.63) showed acceptable reliability given their multidimensional structure. Item‐level CES‐D data were unavailable due to harmonization, but its established validity and expected associations in this sample support appropriate measurement.^26^

### 2.5 Statistical analysis

Descriptive statistics summarized demographic, psychosocial, neighborhood, sleep, and cognitive characteristics. Group comparisons for continuous variables were conducted using independent *t* tests for two‐group comparisons (good vs. poor sleep quality, defined by PSQI > 7) and one‐way analysis of variance (ANOVA) when comparing across more than two categories. Categorical variables were compared using chi‐squared tests or Fisher exact tests where appropriate (i.e., when expected cell counts were < 5). For the analysis, race was categorized into two groups: White and Other races, with the latter category including older adults self‐identified as Black/African American, Hispanic, Asian, or Native American due to limited subgroup sizes.[Table alz70940-tbl-0001]

Generalized linear models (GLMs) were used to examine the associations between sleep quality and cognitive performance across two outcomes: PACC and MoCA. Sleep quality was modeled both as a continuous variable (PSQI global score) and as a categorical variable (PSQI > 7 vs. ≤ 7). Models were adjusted for age, sex, race, and years of education. To test conditional effects, interaction terms were included between sleep and psychosocial burden (CES‐D depressive symptoms or OCSS chronic stress), as well as three‐way interactions among sleep, psychosocial factors, and neighborhood support.

Model fit was assessed using the Akaike information criterion (AIC), the Bayesian information criterion (BIC), and likelihood ratio χ^2^ tests. A reduction of ≥ 2 points in AIC or BIC was considered evidence of improved fit. Regression results are reported as β coefficients with standard errors (SEs), 95% confidence intervals (CIs), and *p* values. Model assumptions were checked using residual and influence diagnostics. All analyses were performed in R (version 4.3.3).

## 3 RESULTS

### 3.1 Sample characteristics

Among 233 adults aged ≥ 65 years, the mean age was 76.1 years (standard deviation [SD] 7.3), 48.9% were women, and 83.7% identified as non‐Hispanic White (Table 1). Participants had a mean of 17.5 years of education (SD 2.14). Mean cognitive scores were 25.0 (SD 3.02) on the MoCA and −0.05 (SD 0.56) on the PACC, and mean CES‐D scores were 7.02 (SD 6.96).

**TABLE 1 alz70940-tbl-0001:** Descriptive statistics of cognitive, psychosocial, and demographic measures in adults aged ≥ 65 years.

	Total (*N* = 233)	Good sleep quality (PSQI ≤ 7, *n* = 173)	Poor sleep quality (PSQI > 7, *n* = 60)	*p*
Demographics				
Age, mean (SD)	76.1 (7.30)	75.9 (7.27)	76.8 (7.41)	0.424
Sex, *n* (%) male	119 (51.1)	94 (54.3)	25 (41.7)	0.123
Female	114 (48.9)	79 (45.7)	35 (58.3)	
Race, *n* (%) White	195 (83.7)	147 (85.0)	48 (80.0)	0.487
Other races	38 (16.3)	26 (15.0)	12 (20.0)	
Education, mean (SD)	17.5 (2.14)	17.5 (2.13)	17.6 (2.16)	0.883
Psychosocial measures				
CES‐D, mean (SD)	7.02 (6.96)	5.78 (5.81)	10.7 (8.66)	<0.001^***^
Neighborhood support, mean (SD)	14.5 (6.65)	14.9 (6.89)	13.5 (5.85)	0.151
OCSS stress, mean (SD)	10.6 (2.72)	10.5 (2.41)	11.1 (3.44)	0.131
Sleep				
PSQI global, mean (SD)	5.47 (3.34)	3.91 (1.86)	9.95 (2.49)	<0.001^***^
Cognitive measures				
RAVLT delay, mean (SD)	0.06 (1.32)	−0.03 (0.51)	0.33 (2.45)	0.066
MoCA total, mean (SD)	25.0 (3.02)	25.1 (3.01)	24.6 (3.07)	0.318
RAVLT immediate, mean (SD)	−0.33 (0.93)	−0.33 (0.96)	−0.34 (0.85)	0.959
Trails A, mean (SD)	0.35 (1.07)	0.32 (1.07)	0.41 (1.06)	0.580
Trails B, mean (SD)	−0.00 (1.12)	0.03 (0.92)	−0.09 (1.57)	0.482
PACC, mean (SD)	−0.05 (0.56)	−0.07 (0.47)	0.03 (0.76)	0.220

Abbreviations: CES‐D, Center for Epidemiologic Studies Depression Scale; MoCA, Montreal Cognitive Assessment; OCSS, Ongoing Chronic Stressors Scale; PACC, Preclinical Alzheimer's Cognitive Composite; PSQI, Pittsburgh Sleep Quality Index; RAVLT, Rey Auditory Verbal Learning Test; SD, standard deviation; Trails, Trail Making Test.

^***^ *p* < 0.001.

For descriptive comparison and visualization, sleep quality was categorized using a PSQI cutoff of > 7. Based on this, 60 participants (25.8%) had poor sleep quality and 173 (74.2%) had good sleep quality. Women comprised a higher proportion of the poor sleep group (58.3%) than the good sleep group (45.7%), whereas racial distribution was similar (80.0% vs. 85.0% non‐Hispanic White; both *p* > 0.10). Participants with poor sleep had higher CES‐D scores (10.7 ± 8.66 vs. 5.78 ± 5.81; *p* < 0.001) and higher PSQI scores (9.95 ± 2.49 vs. 3.91 ± 1.86; *p* < 0.001). Neighborhood support and chronic stress scores did not differ significantly by sleep status (both *p* > 0.10).

MoCA and PACC scores did not differ significantly by sleep status (MoCA: 24.6 ± 3.07 vs. 25.1 ± 3.01, *p* = 0.318; PACC: 0.03 ± 0.76 vs. −0.07 ± 0.47, *p* = 0.220).

### 3.2 Associations among sleep quality, psychosocial burden, and cognitive performance

#### 3.2.1 Sleep quality, chronic stress, and cognitive performance

Chronic stress displayed a similar pattern to depressive symptoms (Table 2). When sleep was considered continuously, the PSQI global by OCSS interaction was negative (β = −0.0305, SE = 0.0119, 95% CI −0.0538 to −0.0072, *p* = 0.011), indicating that higher stress worsened the impact of poor sleep quality on cognitive performance. The three‐way interaction with neighborhood support was positive (β = 0.0018, SE = 0.0007, 95% CI 0.0004 to 0.0032, *p* = 0.014), indicating that higher social support buffered the combined negative effects of poor sleep quality and stress.

**TABLE 2:** Association of neighborhood support, sleep, stress (OCSS), and covariates with cognitive performance (PACC) in older adults.

	Model 1^a^: PSQI category				Model 2^b^: PSQI global score			
Predictor	Estimate	SE^c^	*t*	*p*	Estimate	SE^c^	*t*	*p*
Neighborhood support	−0.002	0.023	−0.106	0.916	0.028	0.049	0.578	0.564
Sleep quality (poor sleep quality)	3.256	0.800	4.069	<0.001^***^				
Sleep quality (PSQI global score, continuous)					0.281	0.135	2.091	0.038^*^
Chronic stress (OCSS)^c^	0.005	0.037	0.143	0.886	0.086	0.074	1.154	0.250
Age (years)	−0.022	0.005	−4.620	<0.001^***^	−0.020	0.005	−3.969	<0.001^***^
Race (other)	−0.347	0.094	−3.710	<0.001^***^	−0.378	0.099	−3.821	<0.001^***^
Sex (female)	−0.066	0.065	−1.017	0.310	−0.059	0.068	−0.869	0.386
Education (years)	0.051	0.015	3.352	<0.001^***^	0.050	0.016	3.162	0.002^**^
Neighborhood support × poor sleep quality	−0.219	0.054	−4.079	<0.001^***^				
Neighborhood support × PSQI global score					−0.016	0.008	−1.881	0.061
Neighborhood support × chronic stress	−0.001	0.002	−0.257	0.798	−0.005	0.004	−1.035	0.302
Poor sleep quality × chronic stress	−0.314	0.068	−4.640	<0.001^***^				
PSQI global score × chronic stress					−0.030	0.012	−2.567	0.011^*^
Neighborhood support × poor sleep quality × chronic stress	0.021	0.004	4.942	<0.001^***^				
Neighborhood support × PSQI global score × chronic stress					0.0018	0.001	2.482	0.014^*^

Abbreviations: AIC, Akaike information criterion; df, degrees of freedom; OCSS, Ongoing Chronic Stressors Scale; PACC, Preclinical Alzheimer's Cognitive Composite; PSQI, Pittsburgh Sleep Quality Index; SE, standard error.

^a^ Model 1 (PSQI Category): residual deviance = 50.21 (df = 220), AIC = 329.29.

^b^ Model 2 (PSQI global score): residual deviance = 55.93 (df = 220), AIC = 354.34.

^c^ Estimates are unstandardized regression coefficients from generalized linear models.

^*^ *p* < 0.05, ^**^ *p* < 0.01, ^***^ *p* < 0.001.

**FIGURE 1:**
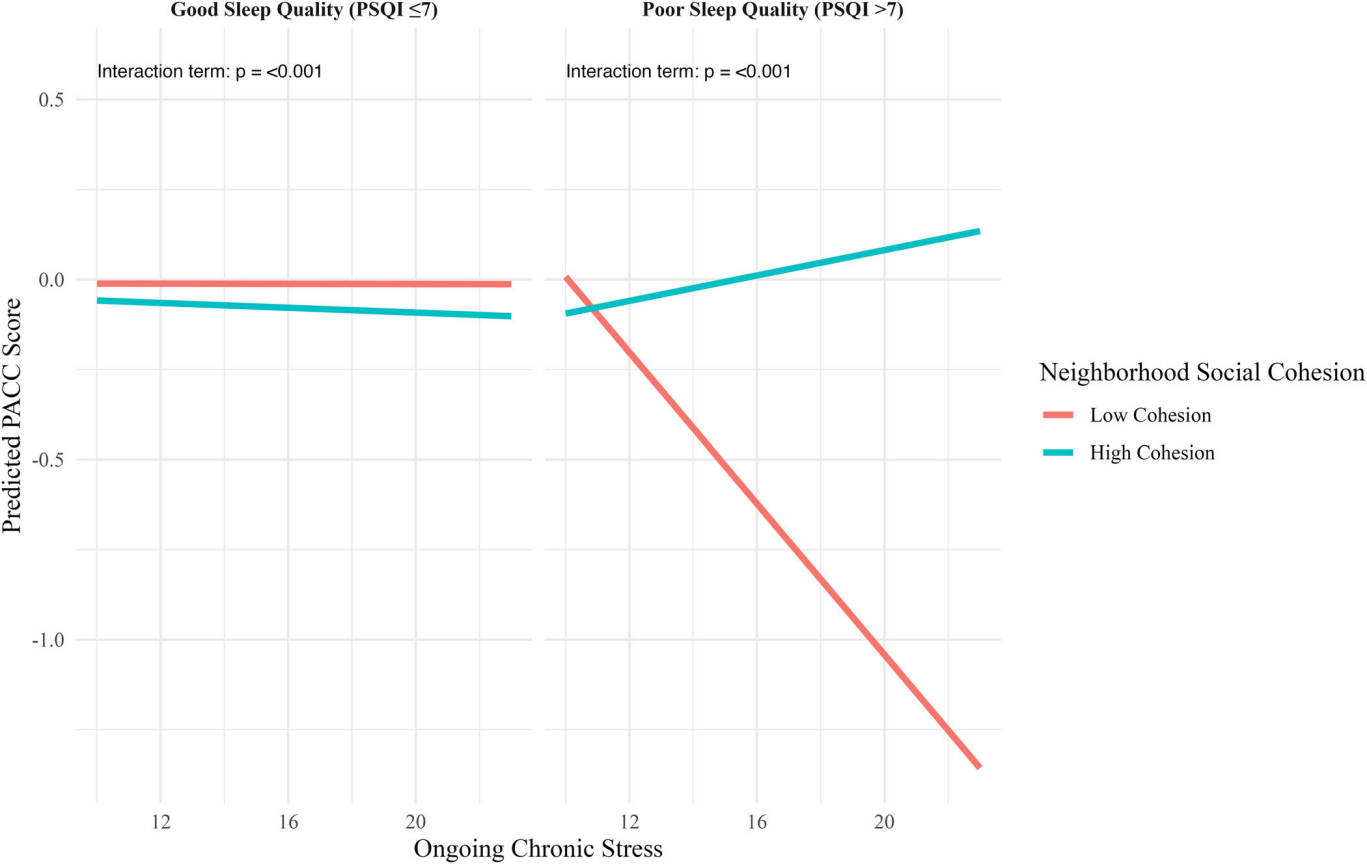
Interaction of sleep quality, neighborhood cohesion, and psychosocial stress in relation to cognitive performance (PACC). Sleep quality: PSQI ≤ 7 = good sleep quality; PSQI > 7 = poor sleep quality; ongoing chronic stress burden: modeled as a continuous variable; neighborhood cohesion: dichotomized using quantile cut points at the 33rd and 67th percentiles (low vs. high); covariates adjusted for age, sex, race, and education; cognitive outcome: PACC; three‐way interaction is significant at *p* < 0.05. PACC, Preclinical Alzheimer's Cognitive Composite; PSQI, Pittsburgh Sleep Quality Index

When sleep was treated categorically, the interaction between poor sleep quality and chronic stress worsened cognitive performance (β = −0.3145, SE = 0.0678, 95% CI −0.4474 to −0.1816, *p* < 0.001), while the three‐way interaction with neighborhood support buffered this effect (β = 0.0210, SE = 0.0042, 95% CI 0.0127 to 0.0293, *p* < 0.001; Table 2). Covariates mirrored similar patterns observed in prior analyses: age was negative, education was positive, and “Other races” were negative, with sex not being associated. These regression patterns are illustrated in Figure 1.

#### 3.2.2 Sleep quality, depressive symptoms, and cognitive performance

**FIGURE 2:**
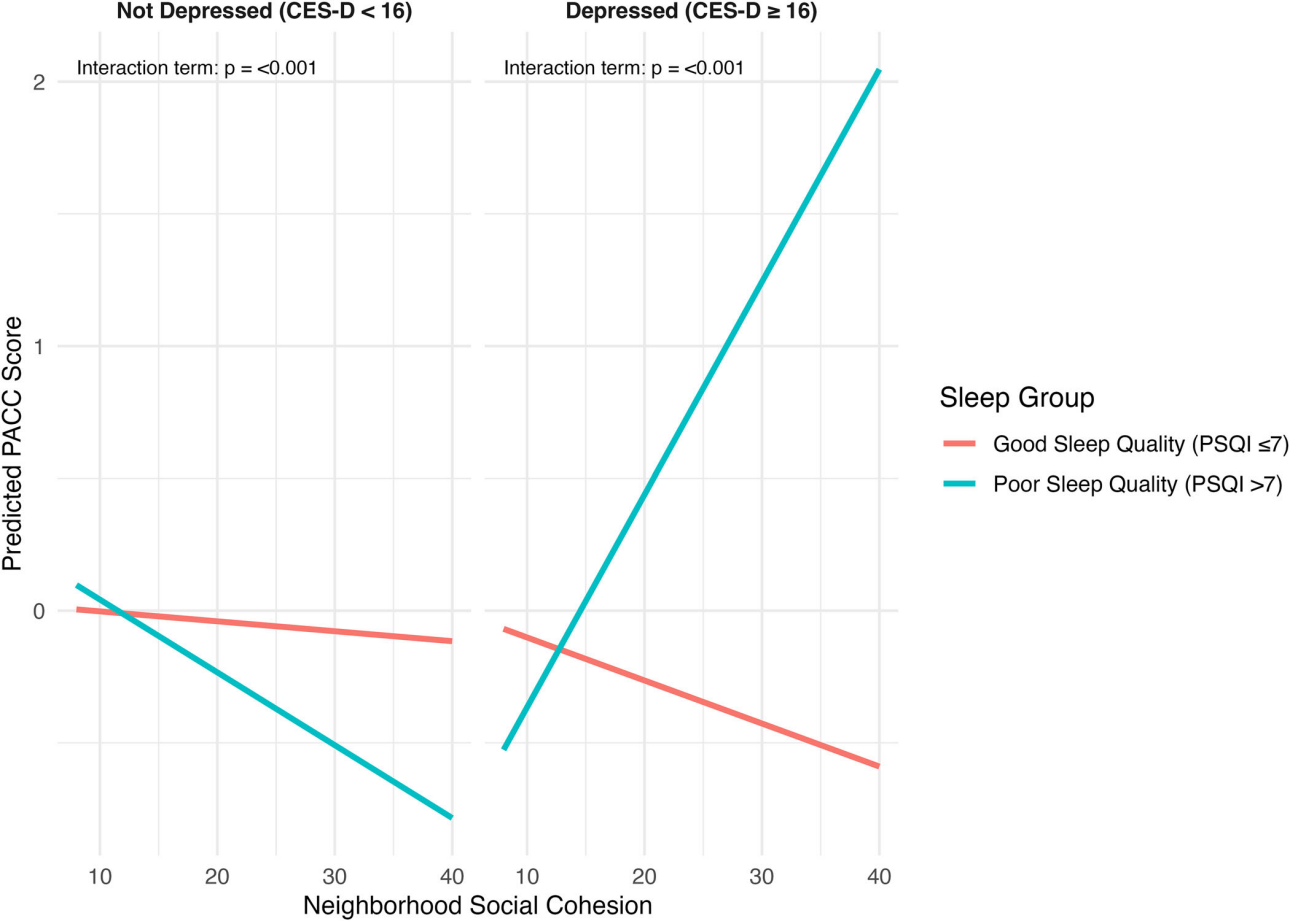
Interaction of sleep quality, neighborhood cohesion, and depression in relation to cognitive performance (PACC). Sleep quality: PSQI ≤ 7 = good sleep quality; PSQI > 7 = poor sleep quality; neighborhood cohesion: modeled as a continuous variable; depressive symptoms: categorized as not depressed (CES‐D] < 16) and depressed (CES‐D ≥16); covariates adjusted for: age, sex, race, and education; cognitive outcome: PACC; three‐way interaction is significant at *p* < 0.05. CES‐D, Center for Epidemiologic Studies Depression Scale; PACC, Preclinical Alzheimer's Cognitive Composite; PSQI, Pittsburgh Sleep Quality Index

Depressive symptoms further shaped the relationship between sleep and cognition (Table 3). With sleep coded categorically, the interaction between poor sleep quality and CES‐D score was negative (β = −0.0865, SE = 0.0242, 95% CI −0.1340 to −0.0390, *p* < 0.001), showing that higher depressive symptoms strengthened the negative association of poor sleep quality with PACC. The three‐way interaction among neighborhood support, poor sleep quality, and CES‐D score was positive (β = 0.0069, SE = 0.0014, 95% CI 0.0042 to 0.0096, *p* < 0.001), suggesting that higher neighborhood support reduced the increasingly negative sleep–depression interaction. Figure 2 illustrates these patterns.

**TABLE 3:** Association of neighborhood support, sleep, depressive symptoms (CES‐D), and covariates with cognitive performance (PACC) in older adults.

	Model 1^a^: PSQI category				Model 2^b^: PSQI global score			
Predictor	Estimate	SE^c^	*t*	*p*	Estimate	SE^c^	*t*	*p*
Neighborhood support	−0.001	0.008	−0.066	0.947	0.009	0.017	0.545	0.586
Sleep quality (poor sleep quality)	0.672	0.294	2.283	0.023^*^				
Sleep quality (PSQI global score, continuous)					0.061	0.047	1.296	0.196
Depressive symptoms (CES‐D score, continuous)^c^	0.002	0.017	0.092	0.927	0.041	0.030	1.337	0.183
Age (years)	−0.019	0.005	−4.079	<0.001^***^	−0.019	0.005	−3.874	<0.001^***^
Race (other)	−0.382	0.094	−4.047	<0.001^***^	−0.373	0.099	−3.775	<0.001^***^
Sex (female)	−0.056	0.066	−0.853	0.394	−0.063	0.069	−0.925	0.356
Education (years)	0.049	0.015	3.141	0.002^**^	0.045	0.016	2.757	0.006^**^
Neighborhood support × poor sleep quality	−0.055	0.022	−2.533	0.012^*^				
Neighborhood support × PSQI global score					−0.004	0.003	−1.233	0.219
Neighborhood support × depressive symptoms (CES‐D)	−0.001	0.001	−0.814	0.417	−0.003	0.002	−1.745	0.082
Poor sleep quality × depressive symptoms (CES‐D)	−0.086	0.024	−3.581	<0.001^***^				
PSQI global score × depressive symptoms (CES‐D)					−0.012	0.004	−2.748	0.007^**^
Neighborhood support × poor sleep quality × depressive symptoms (CES‐D)	0.0069	0.001	4.801	<0.001^***^				
Neighborhood support × PSQI global score × depressive symptoms (CES‐D)					0.0008	0.0003	3.042	0.003^**^

Abbreviations: AIC, Akaike information criterion; CES‐D, Center for Epidemiologic Studies Depression Scale; df, degrees of freedom; PACC, Preclinical Alzheimer's Cognitive Composite; PSQI, Pittsburgh Sleep Quality Index; SE, standard error.

^a^ Model 1 (PSQI category): residual deviance = 49.34 (df = 215), AIC = 323.75.

^b^ Model 2 (PSQI Global Score): residual deviance = 54.33 (df = 215), AIC = 345.62.

^c^ Estimates are unstandardized regression coefficients from generalized linear models.

^*^ *p* < 0.05, ^**^ *p* < 0.01, ^***^ *p* < 0.001.

When sleep was examined as PSQI global, the interaction with depression (CES‐D) score was negative (β = −0.0119, SE = 0.0043, 95% CI −0.0203 to −0.0035, *p* = 0.007), while the three‐way interaction with neighborhood support was positive (β = 0.0008, SE = 0.0003, 95% CI 0.0003 to 0.0014, *p* = 0.003). Across all analyses, age was negatively associated with PACC, education was positively associated, and “Other races” scored lower than White participants (*p* < 0.001 for all).

Poor sleep quality was significantly associated with lower PACC scores in both categorical (PSQI > 7 vs. ≤ 7) and continuous models, with neighborhood support attenuating these effects (Table S1 in supporting information). In contrast, MoCA associations were weaker: PSQI global was negatively associated with MoCA, and only the interaction with neighborhood support was significant, while categorical sleep and higher order interactions with depressive symptoms or stress were not (Tables S2–S4 in supporting information). These findings underscore the greater sensitivity of PACC for detecting sleep–psychosocial associations.

## 4 DISCUSSION

In this cross‐sectional analysis of community‐dwelling older adults, poor sleep quality was consistently associated with lower PACC‐based cognitive performance. Furthermore, the negative influence of poor sleep quality was amplified when combined with higher depressive symptoms or higher stress burden, demonstrating synergistic rather than independent effects of these risks. Importantly, neighborhood support attenuated these negative associations: older adults with stronger neighborhood support exhibited less reduction in PACC‐based cognitive performance, despite poor sleep quality and a high psychosocial burden. These patterns were not evident with MoCA, underscoring the greater sensitivity of PACC‐based cognitive performance. These results suggest that the buffering effect of neighborhood support is most evident under conditions of elevated psychosocial risk, consistent with the exposome perspective that cumulative and interacting exposures shape cognitive trajectories in late life.

Prospective studies have shown that both short and long sleep durations predict faster cognitive deterioration and higher dementia risk.^30^, ^31^, ^32^, ^33^ Meta‐analyses confirm that sleep disturbance is a significant risk factor for incident dementia.^34^, ^35^ Mechanistic research provides biological explanations: reduced slow‐wave sleep decreases the clearance of amyloid beta and tau,^36^ fragmented sleep heightens systemic inflammation,^37^, ^38^ and structural imaging studies reveal hippocampal atrophy and altered white matter connectivity among older adults with poor sleep quality.^39^, ^40^ In line with these findings, our results demonstrate that poor sleep quality was negatively associated with global cognition, supporting the idea that sleep quality is a modifiable factor in late‐life cognitive health.

Although depressive symptoms alone did not show a strong independent association with PACC, their presence magnified the negative influence of poor sleep quality on cognition. In other words, older adults with both poor sleep quality and higher depressive symptoms exhibited disproportionately lower PACC‐based cognitive performance compared to those with only one of these risks. Depression has repeatedly been linked to dementia risk, with meta‐analyses suggesting that late‐life depression nearly doubles the likelihood of developing AD.^41^ Shared biological pathways may explain the synergy: both poor sleep quality and depression contribute to hypothalamic–pituitary–adrenal (HPA) axis dysregulation, elevated cortisol, increased pro‐inflammatory cytokines, and reduced neurotrophic signaling, all of which undermine neuronal survival and function.^42^, ^43^, ^44^, ^45^

Stress exhibited a similar pattern, amplifying the negative association between poor sleep quality and cognitive function. Older adults reporting higher stress combined with poor sleep quality exhibited lower PACC‐based cognitive performance than those with poor sleep quality alone. This pattern is consistent with the concept of allostatic load, which refers to the cumulative physiological wear and tear from chronic stress. ^46^, ^47^, ^48^, ^49^ Chronic stress has been shown to impair hippocampal neurogenesis, shrink dendritic architecture, and compromise synaptic plasticity, ^50^, ^51^ while epidemiological studies link midlife and late‐life stress to higher dementia risk. ^52^, ^53^

Neighborhood support was not strongly associated with cognition when considered alone, but it played a critical buffering role in interaction with other variables. Specifically, greater neighborhood support attenuated the negative associations between poor sleep quality and depressive symptoms, as well as between poor sleep quality and stress. This means that older adults with high psychosocial burden experienced less severe reductions in cognition when they also reported stronger neighborhood support. This pattern is consistent with the stress‐buffering hypothesis, ^54^, ^55^ which proposes that social resources are most effective under adverse conditions. Evidence from cardiovascular, immune, and psychiatric research indicates that supportive environments mitigate the adverse effects of stress on health. ^56^, ^57^, ^58^ The pathways through which neighborhood support exerts its buffering influence are likely multifactorial. Supportive environments may reduce loneliness, enhance coping strategies, and promote engagement in cognitively stimulating activities, thereby positively contributing to cognitive function. ^59^, ^60^ Biological mechanisms also play a role: social support reduces cortisol reactivity and systemic inflammation,^61^ both of which are implicated in sleep disturbance, depression, and cognitive decline.

Two subgroup patterns warrant cautious interpretation. Among older adults with good sleep quality, higher cohesion was associated with slightly lower PACC scores, likely reflecting ceiling effects and limited variability. In the depression models, participants with poor sleep quality and elevated symptoms sometimes showed relatively better PACC‐based cognitive performance under strong support. These findings suggest conditional buffering, potentially reflecting increased engagement with resources or preserved cognitive insight.

Among non‐depressed older adults, those with good sleep quality demonstrated better PACC‐based cognitive performance than those with poor sleep quality, reinforcing sleep quality as a primary determinant of cognition. In contrast, among older adults with higher depression scores, those with poor sleep quality showed relatively higher PACC‐based cognitive performance, particularly when neighborhood support was strong. This is counterintuitive because both poor sleep quality and depression are typically associated with worse, not better, cognition. A likely explanation is that strong neighborhood support helps offset these risks by promoting coping strategies, social engagement, and resource use, which may preserve PACC‐based cognitive performance despite higher symptom reporting. Another possibility is that older adults with greater depressive symptoms may retain stronger cognitive insight, leading them to report more subjective problems even while PACC‐based cognitive performance remains stable. These findings highlight that the buffering role of support becomes visible under conditions of high psychosocial burden, though subgroup patterns should be interpreted cautiously.

This study has several strengths. By examining sleep, depressive symptoms, stress, and neighborhood support together, we captured both independent and interactive effects on cognitive performance. Second, the use of two‐ and three‐way interaction models revealed conditional pathways that would have been missed with main‐effect analyses. Finally, situating these findings within an exposome framework emphasizes their broader public health significance. Several limitations should also be acknowledged. First, the cross‐sectional design precludes causal inference; poor sleep quality and psychosocial burden may contribute to lower cognition, but lower cognition could also lead to perceived difficulties with sleep and stress. Second, all exposures were self‐reported, which may introduce recall or reporting bias. Objective measures for sleep, such as actigraphy and polysomnography, can help identify potential sleep disorders underlying disturbed sleep, as well as alterations in sleep–wake patterns that may point to underlying mechanistic pathways. Additionally, stress‐related biomarkers can provide evidence for the biological pathways through which stress exerts its effects. Third, our sample was relatively small and highly educated, which may limit the generalizability of our findings to socioeconomically diverse populations. Fourth, subgroup anomalies, specifically, lower PACC scores among participants with good sleep quality and higher cohesion, and higher PACC scores among those with poor sleep quality and elevated depressive symptoms under strong support, likely reflect ceiling effects or measurement variability and should be examined in larger, more heterogeneous samples. Fifth, the PSQI and OCSS showed modest internal consistency based on Cronbach alpha, consistent with their multidimensional structure. CES‐D item‐level data were unavailable due to the harmonization process, which only retained total scores.

The implications of these findings are threefold. Clinically, older adults reporting both poor sleep quality and psychosocial burden may represent a high‐risk group for cognitive decline, and identifying neighborhood support could help target resilience factors. Public health interventions that strengthen neighborhood cohesion, such as age‐friendly urban design, community programs, or social prescribing, may provide scalable strategies to mitigate the negative associations between psychosocial risks and cognitive function. From a research perspective, longitudinal studies that integrate objective sleep data, psychosocial assessments, and neighborhood‐level indicators with sensitive cognitive outcomes, such as PACC, will be essential to clarify temporal directions and causal pathways.

In conclusion, poor sleep quality was consistently negatively associated with PACC‐based cognitive performance, and higher depressive symptoms and greater stress burden magnified this negative influence. Neighborhood support buffered these negative associations, attenuating the cognitive risks associated with poor sleep quality and psychosocial burden. While MoCA showed largely null findings, PACC clearly captured these associations, underscoring the value of composite cognitive measures. These findings highlight the importance of considering both psychosocial burdens and contextual supports in cognitive aging, suggesting that neighborhood support is a modifiable factor that can protect cognition in late life.

## CONFLICT OF INTEREST STATEMENT

All authors have disclosed any relevant relationships/activities/interests related to the content of this manuscript. The authors declare no conflicts of interest. Funding sources (NIH/NIA) are detailed in the Acknowledgments section, and no sponsor was involved in study design, data analysis, or manuscript preparation. Author disclosures are available in the supporting information.

## Supporting information



Supporting information

Supporting information
