# Supplementary material for: Neighborhood support as a protective factor for cognition: Associations with sleep, depression, and stress
Source: Alzheimers Dement. 2025 Nov 25;21(11):e70940. doi: 10.1002/alz.70940 (PMC12645227; doi:10.1002/alz.70940)
Supplement: Supplementary file 2 — Supporting information [file ALZ-21-e70940-s001.docx]

**Supplementary Table 1.** Association of Neighborhood Support, Sleep, and Covariates with Cognitive Performance (PACC) in Older Adults.

| **Predictor** | **Estimate** | **SE**^§^ | **t** | **p-value** | **Estimate** | **SE**^§^ | **t** | **p-value** |
| --- | --- | --- | --- | --- | --- | --- | --- | --- |
|  | **Model 1**^†^**: PSQI Category** |  |  |  | **Model 2**^‡^**: PSQI Global Score** |  |  |  |
| Neighborhood support | –0.008 | 0.006 | –1.240 | 0.216 | –0.027 | 0.012 | –2.222 | 0.027^*^ |
| Sleep quality (Poor sleep quality) | –0.570 | 0.178 | –3.195 | 0.002^**^ |  |  |  |  |
| Sleep quality (PSQI global score, continuous) |  |  |  |  | –0.074 | 0.030 | –2.494 | 0.013^*^ |
| Age (years) | –0.018 | 0.005 | –3.702 | <0.001^***^ | –0.018 | 0.005 | –3.600 | <0.001^***^ |
| Race (Other) | –0.375 | 0.099 | –3.806 | <0.001^***^ | –0.374 | 0.101 | –3.714 | <0.001^***^ |
| Sex (Female) | –0.091 | 0.068 | –1.338 | 0.182 | –0.078 | 0.069 | –1.121 | 0.264 |
| Education (years) | 0.048 | 0.016 | 3.013 | 0.003^**^ | 0.051 | 0.016 | 3.132 | 0.002^**^ |
| Neighborhood support × Poor sleep quality | 0.047 | 0.012 | 4.033 | <0.001^***^ |  |  |  |  |
| Neighborhood support × PSQI global score |  |  |  |  | 0.006 | 0.002 | 2.854 | 0.005^**^ |

†. Model 1 (Pittsburgh Sleep Quality Index [PSQI] Category): Residual deviance = 57.39 (df = 224), AIC = 352.33

‡. Model 2 (Pittsburgh Sleep Quality Index [PSQI] Global Score): Residual deviance = 59.71 (df = 224), AIC = 361.49

§. SE = Standard Error; PACC = Preclinical Alzheimer Cognitive Composite. Estimates are unstandardized regression coefficients from generalized linear models.

****P* < 0.001, **P* < 0.01, *P* < 0.05.

**Supplementary Table 2.** Association of Neighborhood Support, Sleep, and Covariates with MoCA Performance in Older Adults.

| **Predictor** | **Estimate** | **SE**^§^ | **t** | **p-value** | **Estimate** | **SE**^§^ | **t** | **p-value** |
| --- | --- | --- | --- | --- | --- | --- | --- | --- |
|  | **Model 1**^†^**: PSQI Category** |  |  |  | **Model 2**^‡^**: PSQI Global Score** |  |  |  |
| Neighborhood support | –0.069 | 0.031 | –2.190 | 0.030^*^ | –0.184 | 0.061 | –3.018 | 0.003^**^ |
| Sleep quality (Poor sleep quality) | –1.438 | 0.921 | –1.561 | 0.120 |  |  |  |  |
| Sleep quality (PSQI global score, continuous) |  |  |  |  | –0.399 | 0.147 | –2.710 | 0.007^**^ |
| Age (years) | –0.178 | 0.025 | –7.098 | <0.001^***^ | –0.184 | 0.025 | –7.393 | <0.001^***^ |
| Race (Other) | –1.204 | 0.506 | –2.381 | 0.018^*^ | –1.203 | 0.502 | –2.397 | 0.017^*^ |
| Sex (Female vs Male) | 0.882 | 0.352 | 2.508 | 0.013^*^ | 0.892 | 0.348 | 2.562 | 0.011^*^ |
| Education (years) | 0.302 | 0.082 | 3.664 | <0.001^***^ | 0.302 | 0.082 | 3.705 | <0.001^***^ |
| Neighborhood support × Poor sleep quality | 0.066 | 0.060 | 1.100 | 0.272 |  |  |  |  |
| Neighborhood support × PSQI global score |  |  |  |  | 0.025 | 0.010 | 2.454 | 0.015^*^ |

†. Model 1 (Pittsburgh Sleep Quality Index [PSQI] Category): Residual deviance = 1542.7 (df = 225), AIC = 1119.6

‡. Model 2 (Pittsburgh Sleep Quality Index [PSQI] Global Score): Residual deviance = 1514.0 (df = 225), AIC = 1115.3

§. SE = Standard Error; MoCA = Montreal Cognitive Assessment. Estimates are unstandardized regression coefficients from generalized linear models.

****P* < 0.001, **P* < 0.01, *P* < 0.05.

**Supplementary Table 3.** Association of Neighborhood Support, Sleep, Depressive Symptoms (CES-D), and Covariates with MoCA Performance in Older Adults.

| **Predictor** | **Estimate** | **SE**^§^ | **t** | **p-value** | **Estimate** | **SE**^§^ | **t** | **p-value** |
| --- | --- | --- | --- | --- | --- | --- | --- | --- |
|  | **Model 1**^†^**: PSQI Category** |  |  |  | **Model 2**^‡^**: PSQI Global Score** |  |  |  |
| Neighborhood support | –0.067 | 0.043 | –1.540 | 0.125 | –0.186 | 0.086 | –2.156 | 0.032^*^ |
| Sleep quality (Poor sleep quality) | –2.223 | 1.573 | –1.413 | 0.159 |  |  |  |  |
| Sleep quality (PSQI global score, continuous) |  |  |  |  | –0.412 | 0.232 | –1.778 | 0.077 |
| Depressive symptoms (CES-D score, continuous) | –0.074 | 0.089 | –0.834 | 0.405 | 0.017 | 0.143 | 0.120 | 0.904 |
| Age (years) | –0.167 | 0.025 | –6.674 | <0.001^***^ | –0.174 | 0.025 | –7.047 | <0.001^***^ |
| Race (Other) | –0.952 | 0.502 | –1.897 | 0.059 | –0.969 | 0.492 | –1.969 | 0.050 |
| Sex (Female) | 0.853 | 0.350 | 2.433 | 0.016^*^ | 0.851 | 0.343 | 2.483 | 0.014^*^ |
| Education (years) | 0.244 | 0.083 | 2.954 | 0.003^**^ | 0.254 | 0.081 | 3.134 | 0.002^**^ |
| Neighborhood support × Poor sleep quality | 0.182 | 0.116 | 1.574 | 0.117 |  |  |  |  |
| Neighborhood support × PSQI global score |  |  |  |  | 0.035 | 0.016 | 2.169 | 0.031^*^ |
| Neighborhood support × Depressive symptoms (CES-D) | 0.001 | 0.005 | 0.165 | 0.869 | –0.006 | 0.009 | –0.670 | 0.504 |
| Poor sleep quality × Depressive symptoms (CES-D) | 0.010 | 0.128 | 0.081 | 0.935 |  |  |  |  |
| PSQI global score × Depressive symptoms (CES-D) |  |  |  |  | –0.011 | 0.020 | –0.537 | 0.592 |
| Neighborhood support × Poor sleep quality × Depressive symptoms (CES-D) | –0.004 | 0.008 | –0.576 | 0.565 |  |  |  |  |
| Neighborhood support × PSQI global score × Depressive symptoms (CES-D) |  |  |  |  | 0.0004 | 0.001 | 0.319 | 0.750 |

†. Model 1 (Pittsburgh Sleep Quality Index [PSQI] Category): Residual deviance = 1420.1 (df = 216), AIC = 1090.1

‡. Model 2 (Pittsburgh Sleep Quality Index [PSQI] Global Score): Residual deviance = 1369.4 (df = 216), AIC = 1081.8

§. SE = Standard Error; MoCA = Montreal Cognitive Assessment; CES-D = Center for Epidemiologic Studies Depression Scale. Estimates are unstandardized regression coefficients from generalized linear models.

****P* < 0.001, **P* < 0.01, *P* < 0.05.

**Supplementary Table 4.** Association of Neighborhood Support, Sleep, Stress (OCSS), and Covariates with MoCA Performance in Older Adults.

| **Predictor** | **Estimate** | **SE**^§^ | **t** | **p-value** | **Estimate** | **SE**^§^ | **t** | **p-value** |
| --- | --- | --- | --- | --- | --- | --- | --- | --- |
|  | **Model 1**^†^**: PSQI Category** |  |  |  | **Model 2**^‡^**: PSQI Global Score** |  |  |  |
| Neighborhood support | –0.029 | 0.129 | –0.223 | 0.823 | –0.056 | 0.254 | –0.220 | 0.826 |
| Sleep quality (Poor sleep quality) | –0.094 | 4.389 | –0.021 | 0.983 |  |  |  |  |
| Sleep quality (PSQI global score, continuous) |  |  |  |  | –0.100 | 0.696 | –0.144 | 0.886 |
| Chronic stress (OCSS) | 0.177 | 0.201 | 0.881 | 0.379 | 0.307 | 0.383 | 0.800 | 0.424 |
| Age (years) | –0.181 | 0.026 | –7.065 | <0.001^***^ | –0.188 | 0.025 | –7.384 | <0.001^***^ |
| Race (Other) | –1.218 | 0.509 | –2.395 | 0.017^*^ | –1.240 | 0.508 | –2.443 | 0.015^*^ |
| Sex (Female) | 0.842 | 0.355 | 2.375 | 0.018^*^ | 0.847 | 0.352 | 2.408 | 0.017^*^ |
| Education (years) | 0.296 | 0.083 | 3.566 | <0.001^***^ | 0.299 | 0.082 | 3.646 | <0.001^***^ |
| Neighborhood support × Poor sleep quality | 0.159 | 0.294 | 0.539 | 0.590 |  |  |  |  |
| Neighborhood support × PSQI global score |  |  |  |  | 0.018 | 0.044 | 0.419 | 0.676 |
| Neighborhood support × Chronic stress (OCSS) | –0.004 | 0.011 | –0.383 | 0.702 | –0.014 | 0.023 | –0.589 | 0.556 |
| Poor sleep quality × Chronic stress (OCSS) | –0.182 | 0.372 | –0.489 | 0.625 |  |  |  |  |
| PSQI global score × Chronic stress (OCSS) |  |  |  |  | –0.032 | 0.061 | –0.514 | 0.607 |
| Neighborhood support × Poor sleep quality × Chronic stress (OCSS) | –0.004 | 0.023 | –0.179 | 0.858 |  |  |  |  |
| Neighborhood support × PSQI global score × Chronic stress (OCSS) |  |  |  |  | 0.001 | 0.004 | 0.256 | 0.798 |

†. Model 1 (Pittsburgh Sleep Quality Index [PSQI] Category): Residual deviance = 1518.8 (df = 221), AIC = 1124.0

‡. Model 2 (Pittsburgh Sleep Quality Index [PSQI] Global Score): Residual deviance = 1500.3 (df = 221), AIC = 1121.2

§. SE = Standard Error; MoCA = Montreal Cognitive Assessment; OCSS = Ongoing Chronic Stressors Scale. Estimates are unstandardized regression coefficients from generalized linear models.

****P* < 0.001, **P* < 0.01, *P* < 0.05
